# Sporadic *PCDH18* somatic mutations in EpCAM-positive hepatocellular carcinoma

**DOI:** 10.1186/s12935-017-0467-x

**Published:** 2017-10-23

**Authors:** Takehiro Hayashi, Taro Yamashita, Hikari Okada, Kouki Nio, Yasumasa Hara, Yoshimoto Nomura, Tomoyuki Hayashi, Yoshiro Asahina, Mariko Yoshida, Naoki Oishi, Hajime Sunagozaka, Hajime Takatori, Masao Honda, Shuichi Kaneko

**Affiliations:** Department of Gastroenterology, Kanazawa University Graduate School of Medical Science, 13-1 Takara-Machi, Kanazawa, Ishikawa 920-8641 Japan

**Keywords:** Hepatocellular carcinoma, EpCAM, PCDH18, Whole exome sequencing, Cancer stem cell

## Abstract

**Background:**

The relationship between specific genome alterations and hepatocellular carcinoma (HCC) cancer stem cells (CSCs) remains unclear. In this study, we evaluated the relationship between somatic mutations and epithelial cell adhesion molecule positive (EpCAM^+^) CSCs.

**Methods:**

Two patient-derived HCC samples (HCC1 and HCC2) were sorted by EpCAM expression and analyzed by whole exome sequence. We measured PCDH18 expression level in eight HCC cell lines as well as HCC1 and HCC2 by real-time quantitative RT-PCR. We validated the identified gene mutations in 57 paired of HCC and matched non-cancerous liver tissues by Sanger sequence.

**Results:**

Whole exome sequencing on the sorted EpCAM^+^ and EpCAM^−^ HCC1 and HCC2 cells revealed 19,263 nonsynonymous mutations in the cording region. We selected mutations that potentially impair the function of the encoded protein. Ultimately, 60 mutations including 13 novel nonsense and frameshift mutations were identified. Among them, *PCDH18* mutation was more frequently detected in sorted EpCAM^+^ cells than in EpCAM^−^ cells in HCC1 by whole exome sequences. However, we could not confirm the difference of *PCDH18* mutation frequency between sorted EpCAM^+^ and EpCAM^−^ cells by Sanger sequencing, indicating that *PCDH18* mutation could not explain intracellular heterogeneity. In contrast, we found novel *PCDH18* mutations, including c.2556_2557delTG, c.1474C>G, c.2337A>G, and c.2976G>T, were detected in HCC1 and 3/57 (5.3%) additional HCC surgical specimens. All four HCCs with *PCDH18* mutations were EpCAM-positive, suggesting that *PCDH18* somatic mutations might explain the intertumor heterogeneity of HCCs in terms of the expression status of EpCAM. Furthermore, EpCAM-positive cell lines (Huh1, Huh7, HepG2, and Hep3B) had lower *PCDH18* expression than EpCAM-negative cell lines (PLC/PRL/5, HLE, HLF, and SK-Hep-1), and *PCDH18* knockdown in HCC2 cells slightly enhanced cell proliferation.

**Conclusions:**

Our data suggest that *PCDH18* is functionally suppressed in a subset of EpCAM-positive HCCs through somatic mutations, and may play a role in the development of EpCAM-positive HCCs.

**Electronic supplementary material:**

The online version of this article (doi:10.1186/s12935-017-0467-x) contains supplementary material, which is available to authorized users.

## Background

Hepatocellular carcinoma (HCC) is a leading cause of cancer death worldwide [1, 2], and it is usually associated with specific risk factors including hepatitis B or C virus infection, high alcohol intake, hemochromatosis, and nonalcoholic fatty liver disease [3]. A typical cancer can harbor thousands of somatic mutations, of which 10–100 might occur in the coding region of genes [4–8]. With the advent of next generation sequencing, recent studies have shown that the HCC genome can contain various somatic mutations, intrachromosomal rearrangements, gene fusions, and focal copy number alterations [9–11]. These studies have also indicated that genes related to two pathways, the p53 and Wnt/β-catenin signaling pathways, are most frequently mutated in HCC. Furthermore, whole genome analysis of the HCC genome has indicated that one of the most frequent mutations identified in HCC is TERT promoter region mutation [12].

HCC is a heterogeneous disease in terms of its morphology, biological behavior, response to treatment, and clinical outcome. Traditionally, this heterogeneity has been explained by cancer cell clonal evolution, and the step-wise acquisition of genetic mutations [13]. However, recent evidence has suggested that HCC may conform to the cancer stem cell (CSC) hypothesis; this hypothesis proposes that a subset of cells with stem cell-like features divide asymmetrically to generate a heterogeneous cell population, and these stem cell-like cells play a fundamental role in tumor maintenance, chemoresistance, and metastasis [14].

In HCC, several CSC markers, including CD133, CD90, CD44, CD24, and CD13 have been identified [15–19]. We have previously demonstrated that HCC subtypes can be defined by the expression of the hepatic stem/progenitor cell markers epithelial cell adhesion molecule (EpCAM) and α-fetoprotein, and that these subtypes correlate with distinct gene expression signatures and patient prognoses [20, 21]. Our previous data also suggested that EpCAM is a marker of liver CSCs, and might be used to enrich a highly tumorigenic and chemoresistant cell population.

In the present study, we sorted EpCAM^+^ and EpCAM^−^ cell populations from fresh HCC specimens and performed whole exome sequencing on the sorted cell populations, to identify the somatic mutations that may explain the intratumor heterogeneity of cells (EpCAM^+^ CSCs and EpCAM^−^ non-CSCs in the same tumor). We further evaluated the identified somatic mutations in independent 57 HCC tissues and EpCAM expression status, to identify the somatic mutations that may explain the intertumor heterogeneity of HCCs (EpCAM-positive and -negative HCCs). Our aim was to examine whether EpCAM expression is associated with specific genetic mutations in EpCAM^+^ CSCs (intratumor heterogeneity) or EpCAM-positive HCCs (intertumor heterogeneity), and to determine whether HCC conforms to the clonal evolution or CSC model.

## Methods

### Cell culture

HuH1, HuH7, HLE, HLF, Hep3B, HEP-G2, SK-Hep-1, and PLC/PRL/5 human liver cancer cell lines were obtained from the Japanese Collection of Research Bioresources (JCRB; Osaka, Japan) or American Type Culture Collection (ATCC; Manassas, VA). Cells were routinely cultured in DMEM supplemented with 10% FBS. Two fresh HCC specimens (HCC1 and HCC2) were obtained and were used for xenotransplantation and to prepare single-cell suspensions for analysis. Primary HCC tissues were dissected and digested in 1 mg/mL type 4 collagenase (Sigma-Aldrich Japan K.K., Tokyo, Japan) solution at 37 °C for 15–30 min. Contaminated red blood cells were lysed with ammonium chloride solution (STEMCELL Technologies, Vancouver, BC, Canada) on ice for 5 min.

### Fluorescence activated cell sorting (FACS)

Cultured cells were trypsinized, washed, and resuspended in Hank’s Balanced Salt Solution (Lonza, Basel, Switzerland) supplemented with 1% HEPES and 2% FBS. Cells were then incubated with antibodies on ice for 30 min. Labeled cells were analyzed by FACS using a FACSCalibur (BD Biosciences, San Jose, CA). The antibodies used were: a FITC-conjugated anti-EpCAM monoclonal antibody (Clone Ber-EP4; DAKO, Carpinteria, CA); an APC-conjugated anti-CD326 (EpCAM) antibody (Miltenyi Biotec K.K., Tokyo, Japan); an APC-conjugated anti-CD90 monoclonal antibody (Clone 5E10; eBioscience, San Diego, CA); an APC-conjugated anti-CD133/2 antibody (Clone 293C3; Miltenyi Biotec K.K.); an APC-conjugated anti-CD44 mouse monoclonal antibody (eBioscience); an APC-conjugated anti-CD13 antibody (eBioscience); and a PE-conjugated anti-CD24 antibody (Miltenyi Biotec K.K.).

### Cell sorting

Cells were trypsinized, washed, and resuspended in Hank’s Balanced Salt Solution supplemented with 1% HEPES and 2% FBS. Cells were then incubated with an APC-conjugated anti-CD326 (EpCAM) on ice for 30 min, and EpCAM positive and negative cells were isolated using a BD FACSAria II cell sorting system (BD Biosciences). In addition, EpCAM^+^ and EpCAM^−^ cells were also sorted for functional studies using an autoMACS pro cell separator and CD326 (EpCAM) microbeads (Miltenyi Biotec K.K.).

### Immunohistochemistry (IHC) analyses

HCC tissue samples were obtained from patients who had undergone radical resection at the Center for Liver Diseases in Kanazawa University Hospital, Kanazawa, Japan. All patients provided informed consent and the tissue acquisition procedures were approved by the Ethics Committee of Kanazawa University. In total, 57 formalin-fixed paraffin-embedded HCC samples, resected between 2008 and 2011, were used for the IHC analyses. IHC was performed using EnVision+ Kits (DAKO, Carpinteria, CA), according to the manufacturer’s instructions. An anti-EpCAM monoclonal antibody (VU-1D9; Oncogene Research Products, San Diego, CA) was used to detect EpCAM expression.

### Spheroid formation assays

For spheroid formation assays, single cell suspensions from HCC1 and HCC2 were generated using FACS and 1.5 × 10^4^ HCC1 cells or 1.0 × 10^4^ HCC2 cells were seeded in 6-well Ultra-Low Attachment Microplates (Corning, Corning, NY). The number of spheroids was determined 21 days after seeding.

### Tumorigenicity in NOD/SCID mice

The protocol for animal procedures was approved by the Kanazawa University Animal Care and Use Committee. Cells were suspended in 200 µL of 1:1 DMEM:Matrigel (BD Biosciences) and were subcutaneously injected into 6-week-old NOD/SCID mice (NOD/NCrCRl-Prkdc^scid^) purchased from Charles River Laboratories, Inc. (Wilmington, MA). The size and incidence of subcutaneous tumors was recorded. For histological evaluation, tumors were formalin-fixed and paraffin-embedded prior to storage.

### Quantitative reverse transcription-PCR analysis

Total RNA was extracted using High Pure RNA Isolation Kit (Roche Diagnostics K.K., Tokyo, Japan) according to the manufacturer’s instructions. The expression of selected genes was determined in triplicate using the 7900 Sequence Detection System (Applied Biosystems, Foster City, CA). Each sample was normalized relative to 18s rRNA expression. The probes used were PCDH18, Hs01556217_m1; CYP2R1, Hs01379776_m1; TECTA, Hs00193706_m1; ITGB8, Hs00174456_m1; CSMD1, Hs00899130_m1; PER1, Hs01092603_m1; ALKBH3, Hs00286731_m1; OSCP1, Hs00376771_m1; and 18s rRNA, Hs99999901_s1 (Applied Biosystems).

### RNA interference

Small interfering RNAs (siRNAs) specific to PCDH18 (#1, HSS122980: #2, HSS122982) and a negative control (12935200) siRNA were designed and synthesized by Invitrogen (Invitrogen, Carlsbad, CA). A total of 2 × 10^5^ cells were seeded into 6-well plates 24 h before transfection. Transfection was performed using Lipofectamine RNAiMAX Transfection Reagent (Invitrogen), according to the manufacturer’s instructions. A total of 20, 40, 60, and 100 pmol/L of siRNAs was used for each transfection in SK-Hep-1, HCC2, HLE, and HLF, respectively.

### DNA extraction and whole exome sequencing

DNA extraction was performed using the QIAamp DNA Mini Kit (Qiagen, Hilden, Germany). The SureSelect Human All Exon V4 Kit (Agilent Technologies, Santa Clara, CA) was used for whole exome capture, and the HiSeq 2000 Sequencing System (Illumina Inc., San Diego, CA) was used for massive parallel sequencing. The sequence reads were mapped against the University of California, Santa Cruz hg19 Genome Browser (http://hgdownload.cse.ucsc.edu/goldenPath/hg19/chromosomes/). Sequence variations, including single nucleotide polymorphisms and insertion/deletions were detected using the Genome Analysis Toolkit software (GATK; Broad Institute, Cambridge, MA). All of the whole exome sequencing and analysis was performed at Riken Genesis (Riken Genesis, Tokyo, Japan). To predict the effect of nonsynonymous single nucleotide substitutions on protein structure, function, and phenotype, we used tools available online, such as SIFT (http://sift.jcvi.org/) [22] and Polyphen2 (http://genetics.bwh.harvard.edu/pph2/) [23].

### DNA extraction and Sanger sequencing

DNA extraction was performed using the QIAamp DNA Mini Kit (Qiagen). PCR Primers were designed by Primer-BLAST (http://www.ncbi.nlm.nih.gov/tools/primer-blast/). Primers are listed in Tables 1 and 2. PCR amplifications were performed using Takara Taq Hot Start Version (Takara, Shiga, Japan), or PrimeSTAR GXL DNA Polymerase (Takara) using a standard application protocol and the manufacturer’s instructions. PCR cleanup was performed using the QIAquick PCR Purification Kit (Qiagen). The cleaned PCR products were sequenced using a BigDyeTerminator v3.1 cycle sequencing KIT (Applied Biosystems). Sequenced products were run on the Life Technologies 3130xl Genetic Analyzer (Applied Biosystems). Electropherograms were visualized and analyzed using Sequence Scanner v2.0 software (Applied Biosystems).

**Table 1:** Primers used for PCR amplification

Gene	Exon number	Primer sequence
*PCDH18*	Exon 1	Forward 5′-TAATCTGGGAAGCAAGGGGAC-3′
		Reverse 5′-ACGACCAAACAAACGCAAGG-3′
	Exon 2	Forward 5′-CACTGTCTCCTGCCTCACTG-3′
		Reverse 5′-ATAGTTGGTAGCTGGCTGCG-3′
	Exon 3	Forward 5′-GGCTGTATCGGATGAGGTGG-3′
		Reverse 5′-CCAGCAGGTCTCTCAGCTTC-3′
	Exon 4	Forward 5′-CAGTGGCTAGTTTCTACACGAC3′
		Reverse 5′-TCACACCTAGTTCTTCCCACG-3′

**Table 2:** Primers used for Sanger sequencing

Gene	Target	Primer sequence
*PCDH18*	Exon 1 #1	5′-GCTAAAGTGTGCATCTTTGCTAC-3′
	Exon 1 #2	5′-CAGCAACACTGCACAAATTGC-3′
	Exon 1 #3	5′-CTTCGGGCTTCCTCCATCTC-3′
	Exon 1 #4	5′-TCAGCCCAGAATCCTTGTCC-3′
	Exon 1 #5	5′-TCTGAGGCAGTGAGCTGAAG-3′
	Exon 2	5′-CACTGTCTCCTGCCTCACTG-3′
	Exon 3	5′-GGCTGTATCGGATGAGGTGG-3′
	Exon 4 #1	5′-CACACTTGCATTGTGTACATACG-3′
	Exon 4 #2	5′-GAAGGCGGTAAGAGACGCTG-3′

### Cell proliferation assays

For cell proliferation assays, single cell suspensions of 2 × 10^3^ cells were seeded in 96-well plates, and cell density was evaluated 48 h after seeding using the Cell Counting Kit-8 (Dojindo Laboratories, Kumamoto, Japan) according to the manufacturer’s instructions.

### Statistical analysis

Different test groups were compared using GraphPad Prism software 6.0 (GraphPad Software, San Diego, CA).

## Results

### EpCAM^+^ cells show CSC features in primary HCCs

**Fig. 1:**
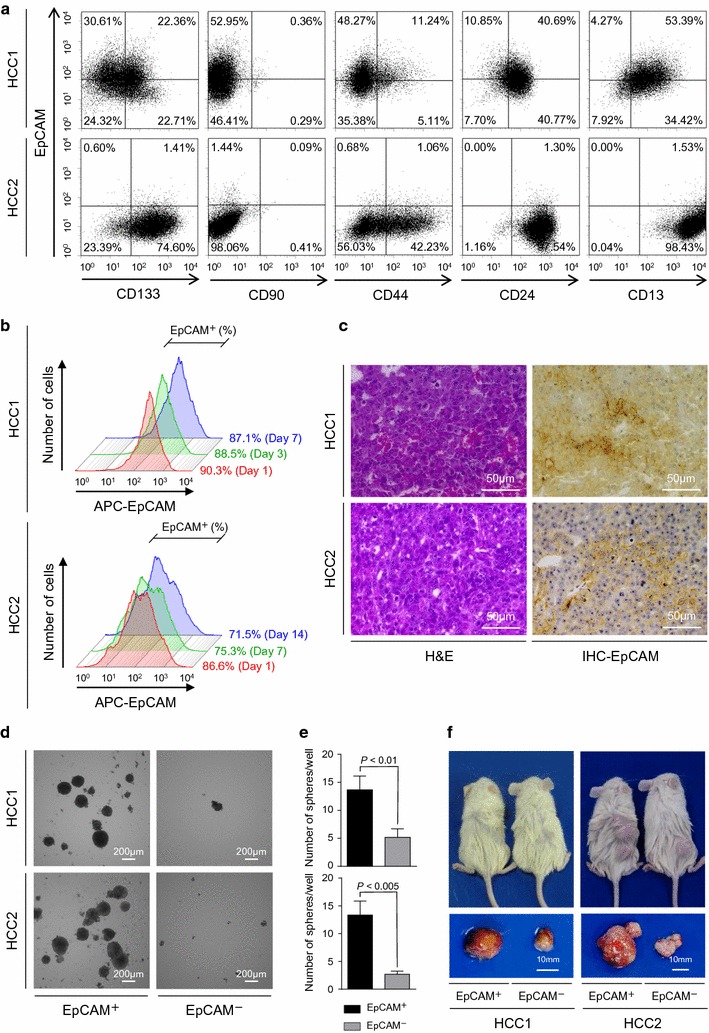
Hepatic stem cell marker expression in HCC1 and HCC2 cells. **a** Flow cytometry of HCC1 and HCC2 cells using fluorescently-labeled antibodies against EpCAM, CD133, CD90, CD44, CD24, and CD13. **b** Flow cytometry of EpCAM^+^ cells using an anti-EpCAM antibody. Figure shows EpCAM^+^ HCC1 cells on days 1, 3, and 7 after cell sorting and EpCAM^+^ HCC2 cells on days 1, 7, and 14 after cell sorting. **c** Histological analysis of EpCAM^+^ HCC1 and HCC2 xenografts. The figure shows hematoxylin and eosin (H&E) staining and anti-EpCAM immunohistochemistry (IHC) staining of the tumors. **d** Representative phase-contrast images of sorted EpCAM^+^ and EpCAM^−^ HCC1 and HCC2 cell spheroids. **e** EpCAM^+^ and EpCAM^−^ HCC1 and HCC2 spheroid formation. Experiments were performed in triplicate. Bars indicate the mean ± standard deviation. **f** Tumorigenic potential of EpCAM^+^ cells. Representative photomicrographs of NOD/SCID mice (upper panel) and subcutaneous tumors (lower panel) from EpCAM^+^ and EpCAM^−^ HCC1 and HCC2 cell xenografts

For this study, we obtained two patient-derived hepatitis C virus-associated HCC samples (HCC1 and HCC2). FACS analysis indicated that both HCC1 and HCC2 contained subsets of EpCAM^+^ cells. HCC1 and HCC2 also contained subsets of cells expressing CD133, CD90, CD44, CD24, and CD13 (Fig. 1a). We enriched EpCAM^+^ and EpCAM^−^ cell populations from HCC1 and HCC2 with > 80% purity using magnetic-activated cell sorting and > 95% purity using FACS. When the sorted EpCAM^+^ HCC cells were cultured for a week, the EpCAM^+^ fraction slightly decreased from 90.3 to 87.1% in HCC1 and 86.6 to 75.3% in HCC2 (Fig. 1b); this is consistent with the ability of EpCAM^+^ cells to divide asymmetrically in vitro and to generate EpCAM^−^ cells. In sorted EpCAM^+^ cells in vivo xenografts, EpCAM^+^ cells also divided asymmetrically and produced tumors with a mixture of both EpCAM^+^ and EpCAM^−^ cells (Fig. 1c). EpCAM^+^ cells also showed strong spheroid forming capacity compared with EpCAM^−^ cells in vitro (Fig. 1d, e), and could efficiently initiate large tumors in NOD/SCID mice (Fig. 1f). All of these results suggest that the EpCAM^+^ cells are CSCs and HCC1 and HCC2 cells conform to the CSC hypothesis.

### Genomic features of patient-derived HCC1 and HCC2 cells

**Fig. 2:**
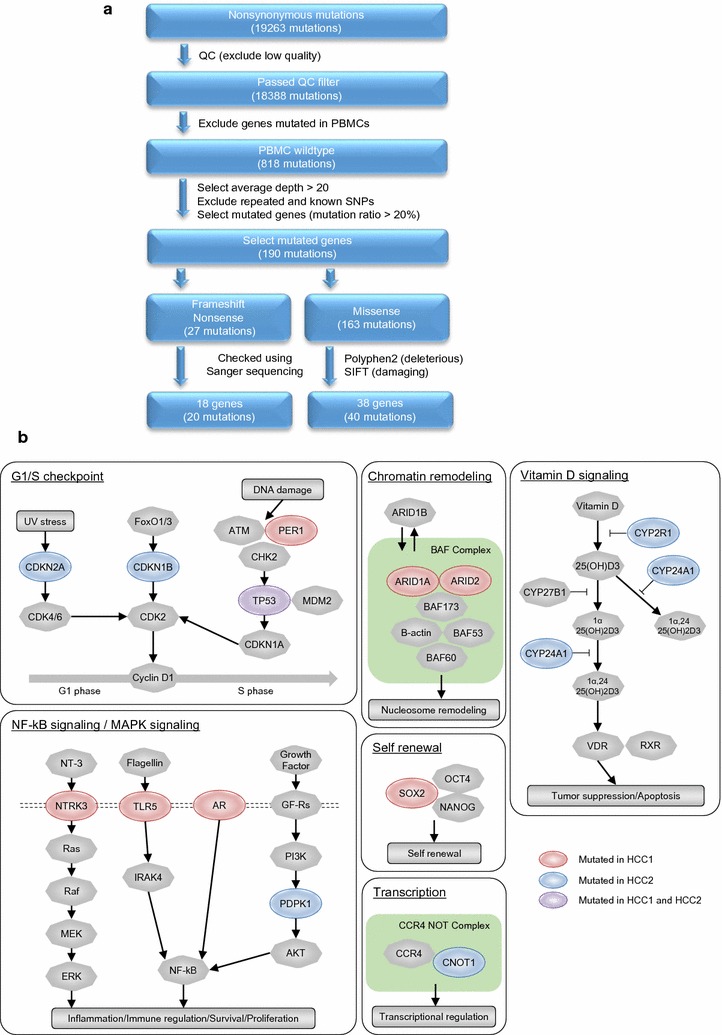
Whole exome sequencing analysis of HCC1 and HCC2. **a** Flowchart of the whole exome sequencing process. Nonsynonymous mutations were extracted from HCC1 and HCC2 cells. Boxes refer to major bioinformatic processes. Variants were filtered for their coding localization, annotation in dbSNP or 1000 genomes, and somatic and functional impairment. **b** The major pathways in which genetic mutations were detected in HCC1 and HCC2 cells. Somatic mutations detected in HCC1 or HCC2 are shown in red or blue, respectively. Mutations detected in both HCC1 and HCC2 are shown in purple

We performed whole exome analysis on the sorted EpCAM^+^ CSCs and EpCAM^−^ non-CSCs in HCC1 and HCC2 cells, obtained from the two primary HCCs, and the patient’s matched peripheral blood mononuclear cells (PBMCs). In total, 19,263 nonsynonymous mutations were identified in the coding region. To isolate the potential somatic mutations that were only present in the EpCAM^+^ CSCs or EpCAM^−^ non-CSCs, and were not present in the matched PBMCs, we performed nucleotide variants analysis by filtering the sequence data according to its qualities, sequence depth, and the available single nucleotide polymorphism database (Fig. 2a). We further selected genetic mutations that potentially impair the function of the encoded protein. Through this, we identified a known *TP53* hotspot mutation in HCC2, c.844C>T; the c.844C>T mutation is reported to a induce p.R282W phenotype [24] and occurs in the second most frequently altered pathway in HCC [10]. In addition, we also identified, and confirmed by Sanger sequencing, 60 potential somatic mutations that affect 56 different genes in HCC1 and HCC2 (see Additional file 1). A novel *TP53* missense mutation, c.767C>T, which would induce p.T256I, was identified in HCC1 (Fig. 2b). In HCC1, we predominantly detected mutations in genes associated with the chromatin remodeling pathway (*ARID1A* and *ARID2*) and NF-κB/MAPK signaling pathway (*NTRK3*, *TLR5*, and *AR*). In contrast, genes associated with the cell cycle G1/S checkpoint (*CDKN1B* and *CDKN2A*) and the vitamin D signaling pathway (*CYP2R1* and *CYP24A1*) were predominantly detected in HCC2. These mutations might reflect frequently mutated pathways, such as *TP53* in EpCAM-positive HCCs [25].

### EpCAM^+^ CSCs and EpCAM^−^ non-CSCs had similar somatic mutation patterns

**Fig. 3:**
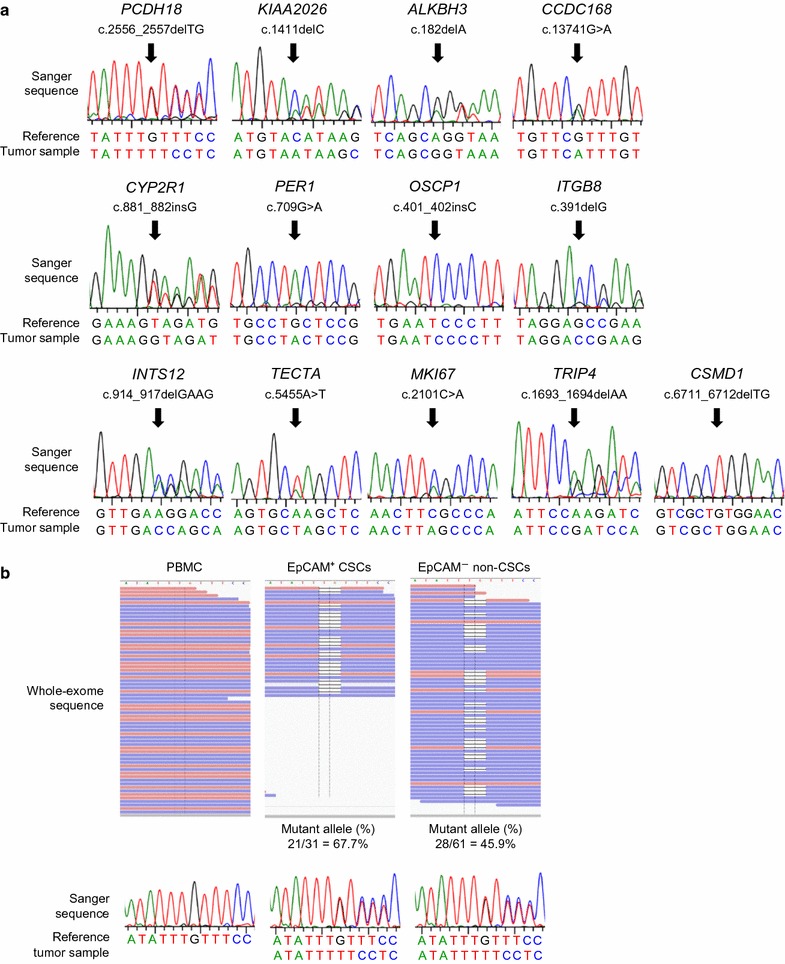
Validation of genetic mutations. **a** Electropherograms of the mutated sequences identified. **b** Frequency of *PCDH18* mutation in PBMCs, EpCAM^+^ CSCs, and EpCAM^−^ non-CSCs. Mutations were evaluated by whole exome sequencing (upper panel) and Sanger sequencing (lower panel)

We proceeded to select frameshift and nonsense mutations that have not previously been published to try to identify potential driver genes that alter protein function in HCC1 and HCC2. We identified 13 novel somatic mutations in HCC1 and HCC2 by Sanger sequencing (Table 3 and Fig. 3a). The number of point mutations as well as their function reported previously were assessed according to the previous publications and are available in Additional file 2 [26–40]. We tried to identify any mutations that were more frequently detected in either the sorted EpCAM^+^ CSCs or EpCAM^−^ non-CSCs in HCC.

**Table 3:** Previously unpublished frameshift and nonsense mutations

Gene	Position	Mutation type	Mutant allele (%) in EpCAM^+^ CSCs fraction	Mutant allele (%) in EpCAM^−^ non-CSCs fraction	Difference (%) EpCAM^+^ (%) − EpCAM^−^ (%)	Sample
*PCDH18*	c.2556_2557delTG	Frameshift	67.7	45.9	21.8	HCC 1
*KIAA2026*	c.1411delC	Frameshift	34.4	19.6	14.8	HCC 1
*ALKBH3*	c.182delA	Frameshift	68.4	54.5	13.9	HCC 1
*CCDC168*	c.13741G>A	Nonsense	33.2	28.9	4.3	HCC 2
*CYP2R1*	c.881_882insG	Frameshift	38.7	38.4	0.3	HCC 2
*PER1*	c.709G>A	Nonsense	100	100	0	HCC 1
*OSCP1*	c.401_402insC	Frameshift	94.3	95.2	− 1	HCC 2
*ITGB8*	c.391delG	Frameshift	74.2	80	− 5.8	HCC 2
*INTS12*	c.914_917delGAAG	Frameshift	25.5	33	− 7.5	HCC 1
*TECTA*	c.5455A>T	Nonsense	60.1	68.2	− 8	HCC 2
*MKI67*	c.2101C>A	Nonsense	23.6	33	− 9.4	HCC 1
*TRIP4*	c1693_1694delAA	Frameshift	28.9	38.5	− 9.6	HCC 2
*CSMD1*	c6711_6712delTG	Frameshift	86	100	− 14	HCC 2

Among them, *PCDH18* was the most candidate gene frequently mutated in sorted EpCAM^+^ CSCs than in EpCAM^−^ non-CSCs. However, this difference was too subtle and not clarified by conventional Sanger sequencing (Fig. 3b).

Furthermore, we could not detect other somatic mutations specific to sorted EpCAM^+^ CSCs or EpCAM^−^ non-CSCs by whole exome sequencing. This indicates that the CSCs and non-CSCs showed similar somatic gene mutation patterns and frequencies at least in HCC1 and HCC2 that follow the CSC hypothesis.

### PCDH18 mutations or loss of expression may be associated with the proliferation in EpCAM-positive HCC

**Fig. 4:**
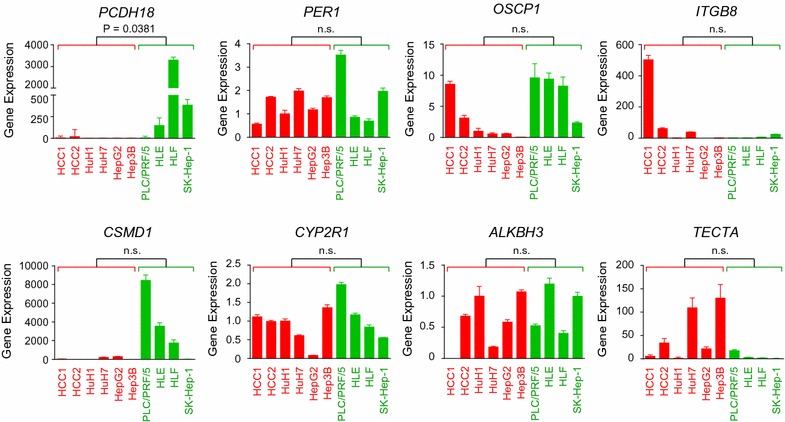
Alteration of gene expression in HCCs. Quantitative reverse transcription-PCR (qRT-PCR) analysis of HCC1 cells, HCC2 cells, and eight HCC cell lines. EpCAM-positive and EpCAM-negative HCC cells are indicated by red and green bars, respectively

Having identified 13 novel mutated genes in the HCC1 and HCC2 cells, we then evaluated their expression to determine whether gene expression is altered by the somatic mutations. We were able to successfully evaluate the expression of 8/13 of the genes in HCC1 cells, HCC2 cells, and eight standard HCC cell lines (Huh1, Huh7, HepG2, Hep3B, PLC/PRF/5, HLE, HLF, and SK-Hep-1; Fig. 4). Interestingly, the level of *PCDH18* gene expression was significantly lower in EpCAM-positive HCC cells (HCC1, HCC2, Huh1, Huh7, HepG2, and Hep3B) compared with EpCAM-negative HCC cells (PLC/PRF/5, HLE, HLF, and SK-Hep-1; P = 0.038; Fig. 4). Although we did not detect *PCDH18* mutations in the EpCAM-positive HCC cell lines, our data suggest that functional suppression of *PCDH18*, through somatic mutation or other mechanisms, may underlie the EpCAM-positive HCC cancer phenotype.

**Fig. 5:**
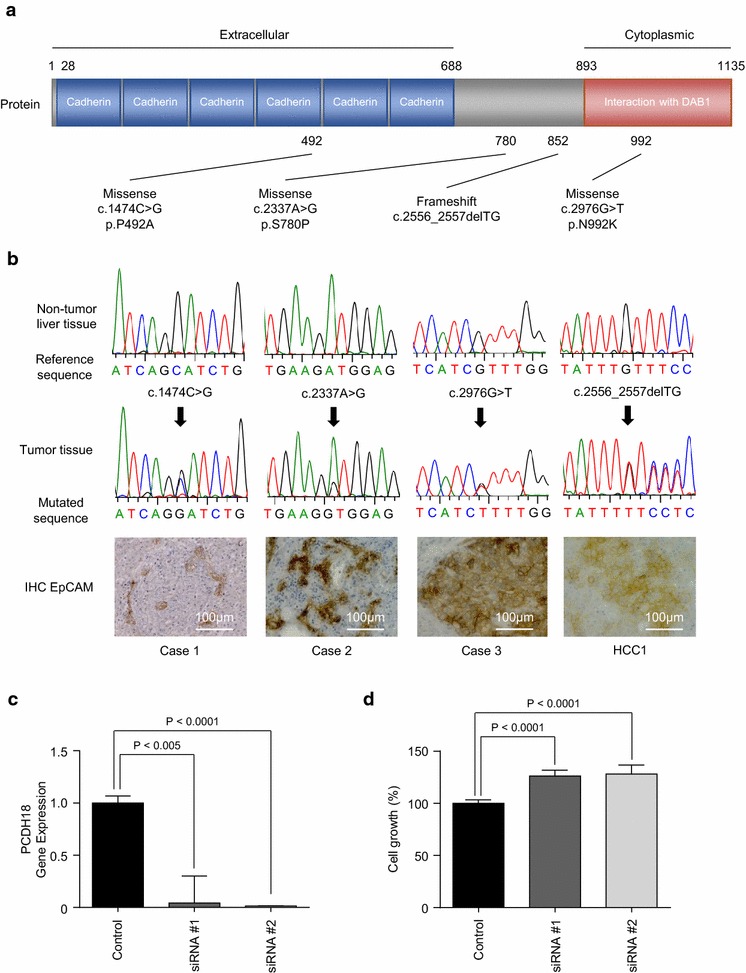
*PCDH18* mutations in HCC. **a** Somatic mutation spectra in 57 HCCs. Nonsynonymous mutations are shown. Functional domains are indicated by colored boxes. **b** Electropherograms of *PCDH18* mutated sequences in HCCs (upper). IHC analysis of HCCs (lower). **c** qRT-PCR analysis of *PCDH18* in HCC2 cells transfected with control or PCDH18-specific siRNAs. **d** Cell proliferation assay of HCC2 cells transfected with control or *PCDH18*-specific siRNAs

Having determined that the level of *PCDH18* gene expression was significantly lower in EpCAM-positive HCC cells, we proceeded to evaluate the frequency of *PCDH18* mutation in 57 additional surgically resected HCC samples. Compared with matched non-tumor liver tissues, 3/57 (5.3%) HCCs harbored *PCDH18* somatic mutations in the tumor tissues, which was validated by Sanger sequencing. We found the novel *PCDH18* mutations including c.1474C>G (p.P492A), c.2337A>G (p.S780P), and c.2976G>T (p.N992K) missense mutations, and a c.2556_2557delTG frameshift mutation in HCC (Fig. 5a). We then evaluated EpCAM expression in the *PCDH18*-mutated HCC cases using IHC analysis (Fig. 5b). In all 57 of the HCC cases, 19/57 (33.3%) HCCs expressed EpCAM, and 3/19 (15.8%) harbored *PCDH18* somatic mutations. In contrast, 38/57 (66.7%) HCCs did not express EpCAM, and using Sanger sequencing, no *PCDH18* somatic mutations were detected in these EpCAM-negative HCCs; this difference was statistically significant (P = 0.033).

**Fig. 6:**
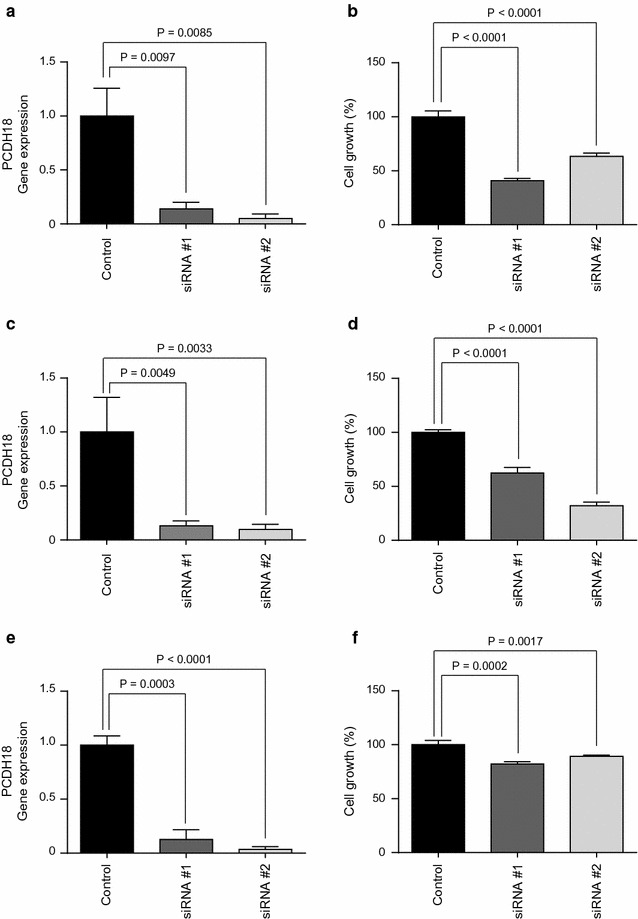
Cell proliferation assay of EpCAM-negative HCC cells. **a** qRT-PCR analysis of *PCDH18* in HLE cells transfected with control or PCDH18-specific siRNAs. **b** Cell proliferation assay of HLE cells transfected with control or *PCDH18*-specific siRNAs. **c** qRT-PCR analysis of *PCDH18* in HLF cells transfected with control or PCDH18-specific siRNAs. **d** Cell proliferation assay of HLF cells transfected with control or *PCDH18*-specific siRNAs. **e** qRT-PCR analysis of *PCDH18* in SK-Hep-1 cells transfected with control or PCDH18-specific siRNAs. **f** Cell proliferation assay of SK-Hep-1 cells transfected with control or *PCDH18*-specific siRNAs

To evaluate the functional role of *PCDH18* in HCC, we transiently knocked down *PCDH18* using small interfering RNAs (siRNAs; *PCDH18* siRNA#1 and *PCDH18* siRNA#2) in HCC2 cells. HCC2 cells were used because *PCDH18* expression was only detectable in EpCAM-positive HCC2 cells, and could not be detected in the other EpCAM-positive HCC cells. *PCDH18* expression was successfully suppressed using *PCDH18*-specific siRNAs compared with control siRNAs (Fig. 5c). Compared with the control, *PCDH18* knockdown slightly enhanced cell proliferation in HCC2 cells (Fig. 5d). In contrast, knockdown of *PCDH18* in EpCAM-negative HLE, HLF, and SK-Hep-1 cells resulted in the inhibition of cell proliferation (Fig. 6a–e). Taken together, our data suggests the different roles of *PCDH18* on cell proliferation between EpCAM-positive and EpCAM-negative HCCs.

## Discussion

It remains to be determined whether intratumor heterogeneity originates from the clonal evolution of tumor cells, with the step-wise acquisition of genetic changes (clonal evolution model), or a balance of self-renewal and differentiation by CSCs, which could potentially be regulated by the microenvironmental niche (CSC model). It is also possible that both models are true to a greater or lesser extent [41–44]. We have postulated that CSCs have a greater potential to acquire genetic mutations than non-CSCs because they are reported to be more resistant to chemo/radiation therapy, and are highly tumorigenic and metastatic. However, in the current study, we found that our two primary HCC cells that follow the CSC model had similar somatic mutation patterns in EpCAM^+^ CSCs and EpCAM^−^ non-CSCs. This suggests that at certain points and conditions in the process of tumorigenesis, CSCs and non-CSCs are genetically similar, and that differences in their tumorigenic/metastatic ability may be conferred by signaling pathways rather than genetic alterations. However, because our data only reflect the exome status of two HCC nodules following the CSC hypothesis, it is possible that HCC CSCs acquire more genetic mutations at different organ sites or after chemo/radiation treatments that may confer a treatment-resistant phenotype. Further studies are required to evaluate the relationship between cancer cell evolution, CSCs, and treatment resistance.

Although we did not detect unique mutations that were enriched in EpCAM^+^ CSCs in our two primary HCC samples, we did identify a number of novel somatic mutations. One of these somatic mutations, *PCDH18* (HCC1), was detected in 3/57 HCCs, and *was* significantly associated with EpCAM-positive HCC. The total *PCDH18* mutation frequency was 5.3%, but in EpCAM-positive HCCs the *PCDH18* mutation frequency was 15.8%. Furthermore, although we did not detect *PCDH18* mutations in HCC cell lines, we did find that *PCDH18* gene expression was suppressed in EpCAM-positive HCC cell lines compared with EpCAM-negative cell lines. These data suggest that a functional loss of *PCDH18*, by genetic mutation or other mechanisms such as epigenetic gene silencing, may be associated with the generation of EpCAM-positive HCCs. Indeed, *PCDH18* knockdown in EpCAM-positive HCC2 cells resulted in a slightly enhanced rate of proliferation, indicating that the requirement for *PCDH18* expression may have been bypassed in EpCAM-positive HCCs; in EpCAM-negative HCC cell lines, *PCDH18* knockdown instead inhibited cell proliferation. Taken together, these data suggest that *PCDH18* may play different roles in EpCAM-positive and EpCAM-negative HCCs.

Protocadherins (PCDHs) are members of the nonclassic subfamily of calcium-dependent cell–cell adhesion molecules, which is part of the cadherin superfamily [45]. The cadherin superfamily is classified into classical cadherins, desmosomal cadherins, and PCDHs. The PCDH family is largely divided into two groups based on their genomic structure: clustered PCDHs and non-clustered PCDHs. Non-clustered PCDHs are further classified into three subgroups: δ1, δ2, and ε.

*PCDH18* belongs to the δ2-PCDH subgroup. Other δ2-PCDHs include *PCDH8*, *PCDH10*, *PCDH17*, and *PCDH19* [46]. *PCDH18* is reported to be expressed in the central nervous system and pharyngeal arches of zebrafish embryos [47], and plays a role in cell adhesion, behavior, and migration in zebrafish development [48]. Although the function of *PCDH18* in humans is unclear, some studies have shown that *PCDH18* deletion may be associated with altered brain development, intellectual disability, and multiple malformations with pulmonary hypertension [26–28, 49, 50]. Significantly, several δ2-PCDH members have been reported to function as tumor suppressor genes. For example, *PCDH8* is genetically or epigenetically silenced in breast cancer [51] and mantle cell lymphoma [52]. The *PCDH10* and *PCDH17* promoter regions are reported to be hypermethylated in uterine cervical cancer [53], head and neck cancer, and some gastrointestinal cancers [54–56]. And our study has shown that a loss of *PCDH18* gene expression may be related to the development of EpCAM-positive HCCs. In contrast, our data indicated the requirement of *PCDH18* expression in EpCAM-negative HCC cell lines. We previously demonstrated that EpCAM^+^ CSCs show epithelial cell feature with highly tumorigenic capacity with activation of Wnt signaling, whereas CD90^+^ CSCs show mesenchymal cell feature with highly metastatic capacity with activation of c-Kit signaling. Furthermore, CD90^+^ CSCs were detected in all EpCAM-negative HCC cell lines [57]. Therefore, it is plausible that *PCDH18* may have a role on maintenance of mesenchymal features of CD90^+^ CSCs. The different role of *PCDH18* gene in different cellular contexts needs to be further evaluated in the future.

## Conclusions

*PCDH18* is mutated and might be functionally abrogated in a subset of EpCAM-positive HCCs. These data illustrated the potential role of *PCDH18* as tumor suppressor, warranting the further exploration of its function in EpCAM-positive HCCs.

## Additional files

**Additional file 1.** Validated nonsynonymous somatic mutations.

**Additional file 2.** The number of point mutations in 300 liver cancers and the function of 13 novel mutated genes.

## Abbreviations

HCC: hepatocellular carcinoma
CSC: cancer stem cell
EpCAM: epithelial cell adhesion molecule
PCDH18: protocadherin 18
FACS: fluorescence activated cell sorting
qRT-PCR: quantitative reverse transcription-polymerase chain reaction
siRNA: small interfering RNA
IHC: immunohistochemistry
